# Platinum Nanoparticles: Green Synthesis and Biomedical Applications

**DOI:** 10.3390/molecules25214981

**Published:** 2020-10-28

**Authors:** Sherif Ashraf Fahmy, Eduard Preis, Udo Bakowsky, Hassan Mohamed El-Said Azzazy

**Affiliations:** 1 Department of Chemistry, School of Sciences & Engineering, The American University in Cairo, AUC Avenue, P.O. Box 74, New Cairo 11835, Egypt; sheriffahmy@aucegypt.edu; 2 School of Pharmacy, University of Hertfordshire-Egypt hosted by GAF, R5 New Garden City, New Administrative Capital AL109AB, Cairo 11835, Egypt; 3 Department of Pharmaceutics and Biopharmaceutics, University of Marburg, Robert-Koch-Str. 4, 35037 Marburg, Germany; eduard.preis@pharmazie.uni-marburg.de

**Keywords:** green synthesis, biosynthesis, platinum nanoparticles, anticancer, antioxidant, antibacterial, antifungal

## Abstract

Platinum nanoparticles (PtNPs) have superior physicochemical properties and great potential in biomedical applications. Eco-friendly and economic approaches for the synthesis of PtNPs have been developed to overcome the shortcomings of the traditional physical and chemical methods. Various biogenic entities have been utilized in the green synthesis of PtNPs, including mainly plant extracts, algae, fungi bacteria, and their biomedical effects were assessed. Other biological derivatives have been used in the synthesis of PtNPs such as egg yolk, sheep milk, honey, and bovine serum albumin protein. The green approaches for the synthesis of PtNPs have reduced the reaction time, the energy required, and offered ambient conditions of fabrication. This review highlights the state-of-the-art methods used for green synthesis of PtNPs, synthesis parameters, and their reported biomedical applications.

## 1. Introduction

Platinum nanoparticles (PtNPs) have gained attention as promising tools for biomedical applications [1,2]. They exhibit unique optical properties that can be optimized by adjusting their sizes and shapes. PtNPs possess a localized surface Plasmon resonance, lead to a distinctive absorption band in the UV/Vis region, which is not displayed by the bulk forms [3,4]. Several physical and chemical approaches have been developed for the fabrication of PtNPs, including lithography, laser ablation, aerosol techniques, sol-gel techniques, co-precipitation, chemical reduction, and photochemical reduction. However, these methods are expensive and involve toxic reagents and synthetic stabilizers that may impact the environment and human health [5,6,7]. Green synthesis approaches that involve eco-friendly biological systems have been developed for the synthesis of PtNPs. Plants and their extracts were used for single-step, cost-effective biosynthesis of PtNPs. Plant extracts harbor various bioactive metabolites that could act as bio-reductants and stabilizers during the green synthesis of PtNPs [8,9,10,11]. In addition to plants, other biological entities were employed for green synthesis of PtNPs, including microbes (such as bacteria, viruses, fungi, and yeast) and marine algae [12,13,14,15,16,17,18,19,20,21,22,23,24,25,26]. The use of microbes as a potential biogenic route for the fabrication of PtNPs has some advantages, including their ease of production and capability to reduce Pt ions via enzymatic activity into the zero-valent Pt while maintaining optimum control over the average particle sizes [27]. Additionally, the use of microbes does not involve the use of toxic synthetic reagents, and many microorganisms operate at ambient temperatures [28]. On the other hand, the use of microbes in the biogenic synthesis of PtNPs involves a complicated and tedious multi-step procedure. Since culturing methods and optimization are very crucial, a sterilized environment should be maintained (to prevent cross-contamination by other microorganisms), and the culture conditions should be optimized for light, temperature, pH, inoculation, and nutrients [29,30]. Algae are other relevant biological entities in the green synthesis of PtNPs. Algae, primitive aquatic microorganisms, are rich in various bioactive compounds and natural reductants. Additionally, they are easily harvested, scalable, and their surfaces possess negative charges facilitating the nucleation and growth of the synthesized nanoparticles. Thus, algae can be utilized for the low-cost large-scale production and tailoring of PtNPs [31,32].

This review covers the recent trends for green synthesis of PtNPs, their characterization, and potential applications in nanomedicine. The green synthetic routes employ plant extracts and various biological entities. The influence of reaction conditions on the synthesis of PtNPs is presented, and their morphology and sizes are compared.

## 2. Green Synthesis of PtNPs Utilizing Plant Extracts

Plant extracts contain diverse primary and secondary metabolites, which could serve as natural reducing and capping agents [13,14,33,34,35,36,37]. Several studies have reported using plant extracts for green synthesis of many metallic nanoparticles (MNPs) [38,39,40]. The plant-mediated biosynthesis of MNPs is a simple and rapid process involving mixing the plant extract with the metal ions solution at an optimized temperature and pH. The nanoparticle generation is indicated by the change in color of the reaction medium [41]. Various factors should be optimized to control the average size, morphology, and surface charge of MNPs, including pH, temperature, and contact time [42,43,44,45,46]. Although the precise mechanisms underlying the green synthesis of MNPs using plant extracts have not been thoroughly investigated yet, a bottom-up mechanism has been proposed [47,48]. This proposed mechanism involves four major steps; (i) initial activation step (bio-reduction) where the metal ions are reduced into their zero-oxidation states [49], (ii) subsequent growth and agglomeration of the small nanoparticles into more substantial and more thermodynamically stable particles, (iii) termination, where stabilization and capping of the MNPs are performed to form nanoparticles of diverse morphologies and average sizes [49,50] and (iv) purification and washing of the MNPs usually by centrifugation [50].

**Figure 1:**
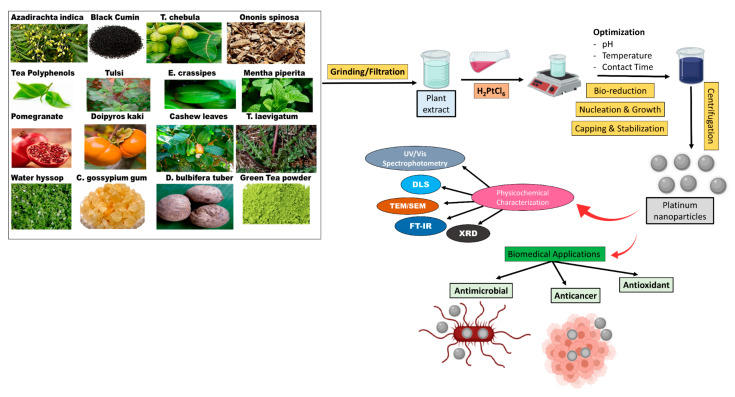
Schematic diagram summarizing the plant species employed in the bio-reduction of PtNPs, parameters optimized, and the techniques employed for characterization of the synthesized nanoparticles.

The average sizes, morphologies, and crystallinity of the synthesized nanoparticles are optimized via controlling the reaction conditions, including pH, temperature, and contact time [51,52]. In addition, different analytical tools were utilized to characterize the fabricated MNPs in general and PtNPs in specific, including UV/Vis spectrophotometry, dynamic light scattering (DLS), scanning electron microscopy (SEM), transmission electron microscopy (TEM), Fourier transform infrared spectroscopy (FTIR), and powder X-ray diffraction (XRD) [53,54,55,56,57,58,59,60]. Figure 1 demonstrates the plant species used for the green synthesis of PtNPs.

### 2.1. Green Synthesis of PtNPs Using Plant Extracts

Plant extracts have been considered a green route and a reliable approach for safe, eco-friendly, and biocompatible PtNPs. The phytosynthesis of PtNPs has been used to replace the current multi-step hazardous synthetic methods. Few studies reported the use of green approaches involving plants and plant extracts. In one study, *Azadirachta indica* leaf broth was incubated with Pt(IV) ions for 1 h at 100 °C. The terpenoids of the *Azadirachta indica* leaf acted as the reducing and capping agents. The generated PtNPs were sonicated for 30 min to enhance the monodispersity of the NPs [61] (Table 1). A recent study reported on the phytosynthesis of PtNPs using *Nigella sativa* (black cumin) seed extract. Pt(IV) ions were stirred with the black cumin extract for two days at 200 rpm and 75 °C [62]. Kumar et al. created a single and simple step for the biosynthesis of PtNPs, employing the fruit extract of *Terminalia chebula*. Many researchers have considered using *Terminalia chebula* in the biosynthesis of MNPs due to its abundance in nature and polyphenolic content. The reaction temperature was maintained at 100 °C for 10 min. The reduction of Pt(IV) ions was mediated by the polyphenols present in the fruit extract [63].

Alshatwi et al. reported on the biosynthesis of PtNPs using tea polyphenols that act as natural reducing agents. Besides, their potential to form chelating complexes with various metal ions allowed them to be used as effective capping agents. The tea polyphenols were mixed with Pt(IV) in a ratio of 1:5 via magnetic stirring and then incubated for 1 h at room temperature [64].

Two studies reported the green synthesis of PtNPs using *Ocimum sanctum* (Tulsi) leave extract. The first synthesis was achieved at 100 °C for 1 h [65]. In contrast, the second one was completed at room temperature by stirring for about 20 min, and the ratio of the plant extract to Pt(IV) ions was kept at 1:9 [66]. The phytosynthesis of PtNPs was done using ethanolic extract of (*Punica granatum)* pomegranate crusts as the reducing agent. Ultrasonication, involving an ultrasonic tip sonicator, was used to mix the pomegranate extract with the metal salt solution, and the mixture was magnetically stirred at room temperature for 24 h incubation period. Pomegranate is an up-and-coming reducing agent because it contains essential oils and resins. Additionally, pomegranate contains various therapeutically active ingredients such as alkaloids, flavonoids, and glycosides [67].

A study conducted by Song et al. reported the green synthesis of PtNPs employing the leaf extract of *Diopyros kaki* (Persimmon). More than 90% of the Pt(IV) ions were bio-reduced into PtNPs using 10% leaf broth concentration at 95 °C for 2–3 h [68]. Sheny et al. biosynthesized PtNPs by mixing dried leaf powder of *Anacardium occidentale* (Cashew) with Pt(IV) ions at 95 °C and pH range 6–9, and the bioreduction reaction was immediate [69]. PtNPs were prepared using the leaf extract of *Bacopa monnieri* (Water hyssop)*,* which was mixed with Pt(IV) ions in a ratio of 1:4 at room temperature [70].

Vinod et al. obtained PtNPs *Cochlospermum gossypium* gum mixed with the metal ions solution at 120 °C using an autoclave (15 psi) and pH 8. This is the only study we found that involved autoclave use to achieve the rapid biosynthesis of PtNPs. Heating and adjusting the pH were involved in activating the glucose, a mild reducing agent, present in the gum extract to achieve controllable bioreduction kinetics [71]. Ghosh et al. employed the tuber extract of *Dioscorea bulbifera* in biosynthesizing PtNPs by conducting the bio-reaction at 100 °C for 5 h [72]. A recent study by Anyik et al. reported the synthesis of PtNPs using the leaf extract of *Eichhornia crassipes* (Water hyacinth). The reaction was carried out at 100 °C for 1 h [79]. In a recent study, PtNPs were biosynthesized by mixing green tea powder extract with Pt (II) ions for 4 h using a magnetic stirrer at 50 °C. The flavonoids present in green tea played a significant role in the bioreduction of Pt(IV) ions owing to their hydroxyl groups [73].

Dobrucka et al. reported on the green synthesis of PtNPs employing *Ononis spinosa radix* extract. Patient ions’ reaction was maintained at 80 °C for 10 h with continuous stirring [74]. Ullah et al. reported the green synthesis of PtNPs utilizing leaf extract of *Maytenus royleanus*. The flavonoids and phenolic compounds present in the leaf extract are responsible for reducing Pt(IV) ions into PtNPs. The reaction temperature was maintained at 90 °C for 3 h with continuous stirring [75].

Yang et al. used the leaf extract of *Mentha piperita* (Peppermint) in biosynthesizing spherical PtNPs by conducting the bio-reduction at 60 °C for 90 min [76]. Tahir et al. developed a facile method for the biosynthesis of spherical PtNPs, employing the plant extract of *Taraxacum laevigatum*. Bioreduction was carried out at 90 °C for 10 min [77]. Finally, eco-friendly PtNPs were fabricated using gum extract of *Prunus x yedoensis.* The reaction conditions were optimized at pH 8 and gum extract concentrations of 7% and 8% for 30 min [78].

### 2.2. Characterization and Biological Activities of PtNPs Prepared Using Plant Extracts

**Figure 2:**
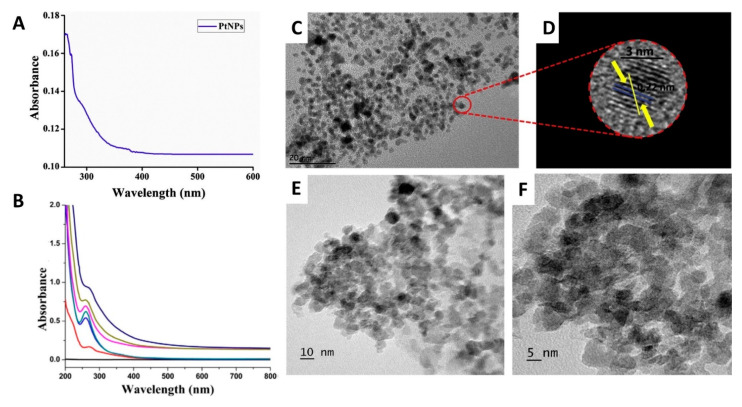
Characterization of PtNPs phytosynthesized utilizing plant extracts using UV/Vis spectrophotometry and TEM. (**A**,**B**) are the UV/Vis spectra of the biosynthesized PtNPs using *Nigella sativa and Mentha piperita*, respectively. (**C**,**D**) are the TEM and the HR-TEM images of the biosynthesized PtNPs using *Nigella sativa.* (**E**,**F**) are the TEM images of the biosynthesized PtNPs using *Mentha piperita* at a scale of 10 and 5 nm, respectively [62,76].

Various analytical tools are utilized in the characterization of PtNPs such as Fourier transform infrared spectroscopy (FT-IR), X-ray diffraction (XRD), transmission electron microscopy (TEM), scanning electron microscopy (SEM), dynamic lights scattering (DLS), and thermal gravimetric analysis (TGA) [61,62,63,64,65,66,67,68,69,70,71,72,73,74,75,76,77,78,79,80,81,82,83,84]. However, dynamic light scattering (DLS), UV/Vis spectrophotometry, and TEM can be used for facile confirmation of the formation of PtNPs (Figure 2).

The formation of *Azadirachta indica* mediated PtNPs was confirmed qualitatively by converting the initial light-yellow color into black, and quantitatively by UV/Vis spectrophotometry. A strong surface Plasmon resonance (SPR) absorption band at 241 nm indicated the formation of the reduced PtNPs. TEM investigations indicated the production of small to large spherical nanoparticles in a size range of 5–50 nm [61].

The PtNPs generated using *Nigella sativa* (black cumin) seed extract were shown to have a spherical shape and an average size of 3.47 nm. Additionally, the formation of PtNPs was confirmed by the appearance of a significant SPR peak at 263 nm (Figure 2A,C,D). The biosynthesized PtNPs were found to have significant anticancer activity against human breast (MDA-MB-231) and cervical (HeLa) cancer cells (IC_50_: 36.86 µg/mL and 19.83 µg/mL; respectively). In addition, the produced NPs showed a pronounced bactericidal activity against Gram-negative and Gram-positive bacteria at concentrations of 100 µg and 500 µg/mL [62].

The *Terminalia chebula* mediated PtNPs were spherical, having an average size of < 4 nm. Besides, the disappearance of the UV/Vis absorption band at 262 nm corresponding to Pt(IV) ions suggested the reduction of Pt(IV) ions [63].

PtNPs prepared using tea polyphenols had flower-shaped morphology and an average size of 30 to 60 nm. In addition, the biosynthesized PtNPs had potent cytotoxic activity, attributed to induction of apoptosis, against cervical human cancer cells (SiHa). The IC_50_ and IC_75_ were 18.34 µg/mL and 11.4 µg/mL, respectively [64].

Two studies reported the green synthesis of PtNPs using *Ocimum sanctum* (Tulsi) leaves extract yielding irregular structured NPs with sizes 23 and 2 nm, respectively [65,66]. PtNPs were characterized by FT-IR, XRD, energy dispersive absorption X-Ray, SEM, and TEM. Additionally, the UV/Vis spectrophotometry was involved as an extra tool to confirm the production of PtNPs. An absorption band was observed at 400 nm, which indicated the formation of PtNPs [66].

The phytosynthesis of monodispersed spherical and cubical PtNPs with an average size of 20 nm was done using ethanolic extract of (*Punica granatum)* pomegranate crusts. The biosynthesized PtNPs were found to have significant cytotoxicity against MCF-7 cancer cells with an IC_50_ of 17.84 µg/mL after 48 h of incubation [67].

PtNPs biosynthesized utilizing the leaf extract of *Diopyros kaki* (Persimmon) had a size range of 2–12 nm. Furthermore, a UV/Vis absorption band was observed at 477 nm, which increased with increasing the Pt concentration, indicating the generation of PtNPs [68]. PtNPs prepared using *Anacardium occidentale* (Cashew) showed irregular rod-shaped morphology. The synthesis of PtNPs was confirmed by the disappearance of the UV/Vis absorption band at 259 nm, corresponding to Pt(IV) ions, and the observation of a continuum at 200 nm suggested the reduction of Pt(IV) ions and the formation of PtNPs. The UV/Vis data showed that the absorbance increases by increasing the quantity of dried leaf powder and decreasing pH [69].

Spherical PtNPs, 5–20 nm, were prepared using the leaf extract of *Bacopa monnieri* (Water hyssop). The formation of the PtNPs was confirmed by the appearance of a UV/Vis absorbance band at 330–380 nm. The biosynthesized nanoparticles were found to have pronounced antioxidant and neuroprotective activities, making them promising candidates for the potential treatment of Parkinson’s disease [70].

Vinod et al. obtained spherical PtNPs of 2.4 nm size using *Cochlospermum gossypium* gum [71]. Ghosh et al. used the tuber extract of *Dioscorea bulbifera* in biosynthesizing spherical PtNPs (2–5 nm). The generated nanoparticles exhibited anticancer activity against human cervical (HeLa) cancer cells. Besides, PtNPs showed antioxidant and pronounced free radical scavenging activity when tested using 2,2-diphenyl-1-picrylhydrazyl, superoxide, nitric oxide, and hydroxyl radicals [72].

The DLS and TEM sizes of the *Eichhornia crassipes* mediated PtNPs were 73.3 and 3.74 nm, respectively, with spherical shapes (it is of note that TEM measures the actual diameter of the PtNPs while DLS measures the diameter in the hydrated state). The disappearance of the UV/Vis absorption band observed at 261 nm corresponding to Pt(IV) ions and the observation of a continuum at 200–300 nm suggested the generation of PtNPs [79]. In another study, the photothermal properties of green tea mediated PtNPs were exploited in cancer therapy. The synthesized PtNPs were found to have a spherical morphology and an average size of 2 nm. The disappearance of the bio-reducer’s absorption band at 320 nm suggested the involvement of the bio-reducer, present in green tea, in reduction of Pt(IV) ions into Pt^0^.

Additionally, the appearance of UV bands in the range of 270–280 suggested the formation of PtNPs. Two human colon cancer cell lines (SW480 and SW620) were used to investigate the anticancer activity of the biosynthesized PtNPs combined with photothermal treatment for 5 min using low-intensity lasers operating at 650 and 808 nm. High cytotoxicity was observed for the biosynthesized PtNPs combined with laser irradiation. The % viability of cancer cells cultured with PtNPs and irradiated with 650 nm laser was found to be 21% and 18% for SW480 and SW620, respectively. While the % viability for cancer cells cultured with PtNPs and irradiated with 808 nm laser was 25% and 22% for SW480 and SW620, respectively. These findings may support future applications of biosynthesized PtNPs in photothermal cancer therapy [73].

Dobrucka et al. reported the green synthesis of spherical and hexagonal PtNPs (4 nm) employing *Ononis spinosa radix* extract. The anticancer activity of the biosynthesized PtNPs was evaluated against A549 cancer cell lines. The maximum cell mortality of 8.8% was observed at an incubation time of 72 h and a concentration of 100 µg/mL of PtNPs [74]. Ullah et al. reported the synthesis of spherical PtNPs of size 5 nm utilizing leaf extract of *Maytenus royleanus*. The appearance of the UV/Vis absorption SPR band at 282 nm suggested the generation of PtNPs. The cytotoxic activity of the prepared PtNPs was evaluated against the A549 cancer cell line. Exposure of A549 cancer cells to increasing concentrations of PtNPs for 24 h has resulted in reduced cell viability, damage to cellular morphology, and reduction in cell number in a dose-dependent manner [75]. Additionally, the PtNPs were found to be biocompatible with normal cells. These findings support the possible development of potent and selective anticancer drugs at low cost [75].

Yang et al. used the leaf extract of *Mentha piperita* (Peppermint) in biosynthesizing spherical PtNPs (54 nm) with surface charge of −50.1 mV, indicating high stability. The appearance of a UV/Vis absorption band at 272 nm indicated the formation of the PtNPs (Figure 2B,E,F). The anticancer activity of the biosynthesized PtNPs was tested against the human colon cancer cell line (HCT116). PtNPs reduced tumor cells’ viability at lower concentrations with an IC_50_ value of 20 µg/mL [76]. The PtNPs created by Tahir et al. employing the plant extract of *Taraxacum laevigatum* were spherical in size range of 2–7 nm. The UV/Vis absorption band’s appearance at 283 nm suggested the formation of PtNPs [77]. The bactericidal activity of the greenly synthesized PtNPs was evaluated against Gram-positive bacteria (*Bacillus subtilis*) and Gram-negative bacteria (*Pseudomonas aeruginosa*). The findings revealed that the PtNPs exhibited significant antibacterial activity against both strains (zones of inhibitions were 15 (± 0.5) mm and 18 (± 0.8) mm for *P. aeruginosa* and *B. subtilis*, respectively), making them promising antibiotics that could overcome bacterial resistance [77]. Finally, spherical PtNPs (10–50 nm) were fabricated using gum extract of *Prunus x yedoensis.* The appearance of the UV/Vis absorption band at 277 nm suggested the creation of the PtNPs. The antifungal activity of the biosynthesized PtNPs was investigated against *Colletotrichum acutatum* and *Cladosporium fulvum* exhibiting 15 mm and 18 mm zones of inhibition at concentrations of 4 and 8 µg/well, respectively [78].

## 3. Green Synthesis of PtNPs Using Miscellaneous Biological Entities

Few studies have reported the synthesis of PtNPs utilizing biological entities other than plants (Table 2). In one study, quail egg yolk, containing peroxidase as a reducing agent, was mixed with Pt(IV) ions and then stirred at 100 rpm for 4 h at 20 °C and pH 6 [80]. The temperature was kept at 20 °C to avoid protein denaturation. The formation of the PtNPs (7–50 nm) was confirmed by the appearance of a sharp UV/Vis peak at 329 nm [80].

Another study exploited *Streptomyces* bacterial strains for the green synthesis of PtNPs. The PtNPs were generated by incubating the bacterial strain with Pt(IV) ions at 50 °C for 24 h. The bio-reduction of the PtNPs was proposed to be mediated by NAD-dependent chloride reductase (the electron shuttle enzymatic metal reduction process). The formation of PtNPs was confirmed by the appearance of a symmetric UV/Vis peak at 262 nm. The biosynthesized PtNPs exhibited a spherical morphology, a zeta potential of −18.4 mV (indicating the stability of the NPs), and a size range of 20–50 nm. The PtNPs exhibited significant cytotoxic activity against human breast cancer cell lines (MCF-7) with IC_50_ of 31.2 µg/mL, which supports further investigation of the potential use of biogenic PtNPs in breast cancer therapy [81]. *Acinetobacter calcoaceticus,* sulfate-reducing bacteria, were involved in the green synthesis of cuboidal PtNPs in size range of 2–3.5 nm at pH 7.0, reaction temperature of 30 °C, and contact time of 24 h [82].

Sheep milk was utilized for the rapid and eco-friendly biosynthesis of spherical PtNPs of an average size of 9 nm and zeta potential of −31 mV. The sheep milk was mixed with Pt(IV) ions and incubated in a rotary shaker (200 rpm) at room temperature for 3 h. The bio-reduction of Pt(IV) ions and the formation of PtNPs was confirmed by the appearance of the UV/Vis peak in the range of 278–284 nm [83]. Highly crystalline Pt nanowires of average size of 2.2 nm were biosynthesized, employing aqueous honey solutions at 100 °C for 2 and 4 h [84]. The globular protein bovine serum albumin (BSA) was used in the green synthesis of spherical PtNPs in a size range of 10–30 nm. The biosynthesis was achieved by adding the Pt(IV) ions to the BSA solution under magnetic stirring at a pH of 3.2 and a temperature of 37 °C for 24 h [85].

Two studies reported on the green synthesis of PtNPs using the aqueous extract of the Indian brown seaweed *Padina gymnospora* yielding truncated octahedral and spherical PtNPs with sizes range 5–50 and 20–35 nm, respectively [86,87]. The first green synthesis was achieved by vigorous stirring at room temperature for 10 min [86], while the second was completed after 3 h incubation at 50 °C [87]. Both studies, PtNPs were characterized using FT-IR, XRD, Energy Dispersive X-ray spectroscopy, SEM, and TEM. In the first study, the disappearance of the peak at 320 nm suggested the formation of PtNPs [86]. The truncated octahedral PtNPs showed maximum bactericidal activity against *Escherichia coli* (15.6 mm, the zone of inhibition), *Lactococcus lactis* (14.8 mm, the zone of inhibition), and *Klebsiella pneumoniae* (14.4 mm, the zone of inhibition) [86]. They were found biocompatible to red blood cells with no observed hemolytic activity [86]. The spherical PtNPs had anticancer activity against A549 lung carcinoma cells with IC_50_ of 19.3 µg/mL and remarkable cytotoxicity [87].

Finally, the fungus *Fusarium oxysporum* was utilized in the green synthesis of spherical PtNPs in the size range of 15–30 nm by shaking the fungal extract with Pt(IV) ions at 200 rpm for 96 h at room temperature [88]. Table 3 summarizes the UV/Vis absorption parameters of the PtNPs biosynthesized using different biological sources.

Green synthesis of PtNPs using different biological systems is affected by critical parameters such as temperature, reaction time, the ratio of metal ions to plant extract, and pH. These parameters control the rate and yield of the PtNPs, their size, shape, and stability. For instance, the biosynthesis rate of *Azardica Indica* mediated PtNPs was directly proportional to the reaction temperature [61]. At a reaction temperature of 25 °C, only 20% of Pt(IV) ions were reduced into PtNPs using *Doipyros kaki*, while at a temperature of 95 °C, 100% of Pt(IV) ions were reduced. Additionally, the biosynthesis rate increased with increasing the *Doipyros kaki* leaf extract concentration where 10% and 100% of PtNPs were formed at leaf concentrations of 5% and 10%, respectively. Additionally, increasing the biosynthesis’s reaction temperature from 25 °C to 95 °C has decreased the size of PtNPs from 12 to 5 nm [68]. Velmurugan et al. found that higher yields of PtNPs were achieved at *Prunus x yedoensis* gum extract concentrations of 7% and 10%. Adjusting the reaction pH is an important factor in increasing both the stability and yield of synthesized PtNPs. For instance, the stability and the UV-Vis absorbance in the *Anacardium occidentale* mediated PtNPs were increased at a pH range of 6–8 [69]. Size optimization of PtNPs is a crucial factor that significantly influences the clinical use of PtNPs in various biomedical applications. PtNPs with an average size of 8 nm exhibited negligible systemic adverse effects, while those with an average size of 1 nm possessed toxic effects against renal cells in a dose-dependent manner. Another study reported the cytocompatibility of PtNPs to the Neuro 2 cell line, while other sizes caused remarkable neurotoxicity. Simultaneously, a study conducted by Konieczny et al. reported the safety of PtNPs with an average size of 57 nm to keratinocytes compared to smaller sizes [89,90].

## 4. Conclusions and Future Directions

In this review, state-of-the-art studies for green synthesis of PtNPs using various biological entities, including plant extracts, algae, microorganisms, egg yolk, sheep milk, and bovine serum albumin are presented. The green synthesis is mainly conducted under ambient conditions and short reaction times. Critical parameters such as temperature, contact time, rate of adding biological entities, and pH must be optimized during the biosynthesis to control the average size and shape of the PtNPs, which will influence their possible use in various biomedical applications. Plants and other biological entities are also rich in different bioactive compounds, which may be deposited onto the PtNPs during synthesis. PtNPs capped with bioactive compounds could be used as promising antibacterial, anticancer, and antifungal agents. Despite the endeavors to develop biosynthesized PtNPs for potential use in nanomedicine, many challenges still exist. As for other MNPs, additional in vitro and animal studies are needed to understand clearance mechanisms of biosynthesized PtNPs, their influence on the immune system and antioxidant activity, and other long-term effects.

## Figures and Tables

**Table 1:** Green synthesis of PtNPs using plant extracts.

Plant	Part Used	Reaction Conditions	Average Size (nm)	Shape	Biomedical Application	Ref.
*Azadirachta indica*	Leaf extract	100 °C; 60 min	5–50	Spherical	-	[61]
*Nigella sativa* (Black cumin)	Seeds	75 °C; 2 days	3.47	Spherical	Anticancer activity against human breast (MDA-MB-231) and cervical (HeLa) cancer cells Bactericidal effect against Gram-negative and Gram-positive bacteria	[62]
*Terminalia chebula*	Fruit extract	100 °C; 10 min	<4	Nearly spherical	-	[63]
Tea polyphenol	-	Room temperature 1 h	30–60	Flower-shaped	Anticancer activity against cervical human cancer cells (SiHa)	[64]
*Ocimum sanctum* (Tulsi)	Leaf extract	100 °C; 1 h	23	Irregular structure	-	[65]
*Ocimum sanctum* (Tulsi)	Leaf extract	Room temperature. Plant extract: Pt ions (1:9) >20 min	2	Irregular structure	-	[66]
*Punica granatum* (Pomegranate)	Crusts	Room temperature Ultrasonication 24 h	20.12	Spherical and cubes	Antiproliferation effect and enhanced apoptosis against human breast adenocarcinoma cell line (MCF-7)	[67]
*Doipyros kaki* (Persimmon)	Leaf extract	95 °C; 2–3 h; Leaf broth concentration: >10%	2−12	Spheres and plates	-	[68]
*Anacardium occidentale* (Cashew)	Leaf extract	95 °C; pH 6–9	-	Irregular rod shaped	-	[69]
*Bacopa monnieri* (Water hyssop)	Leaf extract	Room temperature	5–20	Spherical	Antioxidant and free radical scavenger Neuroprotective	[70]
*Cochlospermum gossypium*	Gum	120 °C; pH 8	2.4	Spherical	-	[71]
*Dioscorea bulbifera*	Tuber extract	100 °C; 5 h	2–5	Spherical	Anticancer activity against human cervical (HeLa) cancer cells Antioxidant	[72]
*Eichhornia crassipes* (Water hyacinth)	Leaf extract	90 °C; 1 h	TEM: 3.74 DLS: 73.3	Spherical	-	[73]
*Green tea*	Powder extract	50 °C; 4 h	2	Spherical	Anticancer activity against two human colon cancer cell lines (SW480 and SW620)	[73]
*Ononis spinose*	Radix extract	80 °C; 10 h	4	Spherical and hexagonal	Anticancer activity against A549 cancer cell lines	[74]
*Maytenus royleanus*	Leaf extract	90 °C; 3 h	5	Spherical	Anticancer activity against A549 cancer cell lines Biocompatible	[75]
*Mentha piperita* (Peppermint)	Leaf extract	60 °C; 2 h	54.3	Spherical	Anticancer activity against human colon cancer cells (HCT116)	[76]
*Taraxacum laevigatum*	Plant extract	90 °C; 10 min	2–7	Spherical	Bactericidal activity against Gram-positive bacteria (*Bacillus subtilis*) and Gram-negative bacteria (*Pseudomonas aeruginosa*)	[77]
*Prunus x yedoensis*	Gum extract	pH 8; Gum extract concentrations of 7% and 8% 30 min	10–50	Spherical	Antifungal against *Colletotrichum acutatum* and *Cladosporium fulvum*	[78]

**Table 2:** Biosynthesis of PtNPs using various biological entities.

Biological Entity	Reaction Conditions	Average Size (nm)	Shape	Biomedical Application	Ref.
Quail egg yolk	20 °C; pH 6; 4 h	7–50	Spherical	-	[80]
*Streptomyces species* (Gram-positive bacteria))	50 °C; 24 h	20–50	Spherical	Anticancer activity against human breast cancer cell lines (MCF-7)	[81]
*Acinetobacter calcoaceticus bacteria*	30 °C; pH 7; 24 h	2–3.5	Cuboidal	-	[82]
*Sheep milk*	room temperature for 3 h.	9	Spherical	-	[83]
*Honey*	100 °C for 2 and 4 h	2.2	Nanowires	-	[84]
*Globular protein bovine serum albumin (BSA)*	37 °C; pH 3.2 for 24 h	10–30	Spherical	-	[85]
*Padina gymnospora* *(brown algae)*	room temperature for 10 min	5–50	Truncated octahedral	Bactericidal activity against *Escherichia coli, Lactococcus lactis*, and *Klebsiella pneumoniae*	[86]
*Padina gymnospora* (brown algae)	50 °C; 3 h	20–35	Spherical	Anticancer activity against A549 lung carcinoma cells	[87]
*Fusarium oxysporum fungus*	Room temperature for 96 h	15–30	Spherical	-	[88]

**Table 3:** UV/Vis absorption bands for the PtNPs synthesized using various biological entities.

Plant	Peak Appeared (nm)	Peak Disappeared (nm)	Reference
*Azadirachta indica*	241 *	-	[61]
*Nigella sativa*	263	-	[62]
*Terminalia chebula*	-	262 **	[63]
*Ocimum sanctum* (Tulsi)	400	-	[66]
*Doipyros kaki* (Persimmon)	477	-	[68]
*Anacardium occidentale* (Cashew)	200	259 **	[69]
*Bacopa monnieri* (Water hyssop)	330–380	-	[70]
*Eichhornia crassipes* (Water hyacinth)	200–300 (continuum)	261 **	[79]
Green tea powder extract	-	320 ***	[73]
*Ononis spinosa*	200–300 (broad continuum)	265	[74]
*Maytenus royleanus*	282	-	[75]
*Mentha piperita*	272	-	[76]
*Taraxacum laevigatum*	283	-	[77]
*Prunus x yedoensis*	277	-	[78]
Quail egg yolk	329	-	[80]
*Padina gymnospora*	-	320	[81]
*Streptomyces species*	262	-	[83]
Sheep milk	278–284	-	[84]

* Indicate the formation of the reduced PtNPs. ** Corresponding to Pt(IV) ions. *** Corresponding to the bio-reducer within the green tea.
